# Emerging and Re-emerging Vector-Borne Infectious Diseases and the Challenges for Control: A Review

**DOI:** 10.3389/fpubh.2021.715759

**Published:** 2021-10-05

**Authors:** Bayissa Chala, Feyissa Hamde

**Affiliations:** Department of Applied Biology, School of Applied Natural Science, Adama Science and Technology University, Adama, Ethiopia

**Keywords:** emerging infectious diseases, re-emerging infectious diseases, climate change, zoonotic diseases, vector-borne infectious diseases

## Abstract

Vector-borne emerging and re-emerging diseases pose considerable public health problem worldwide. Some of these diseases are emerging and/or re-emerging at increasing rates and appeared in new regions in the past two decades. Studies emphasized that the interactions among pathogens, hosts, and the environment play a key role for the emergence or re-emergence of these diseases. Furthermore, social and demographic factors such as human population growth, urbanization, globalization, trade exchange and travel and close interactions with livestock have significantly been linked with the emergence and/or re-emergence of vector-borne diseases. Other studies emphasize the ongoing evolution of pathogens, proliferation of reservoir populations, and antimicrobial drug use to be the principal exacerbating forces for emergence and re-emergence of vector-borne infectious diseases. Still other studies equivocally claim that climate change has been associated with appearance and resurgence of vector-borne infectious diseases. Despite the fact that many important emerging and re-emerging vector-borne infectious diseases are becoming better controlled, our success in stopping the many new appearing and resurging vector-borne infectious diseases that may happen in the future seems to be uncertain. Hence, this paper reviews and synthesizes the existing literature to explore global patterns of emerging and re-emerging vector-borne infections and the challenges for their control. It also attempts to give insights to the epidemiological profile of major vector-borne diseases including Zika fever, dengue, West Nile fever, Crimean-Congo hemorrhagic fever, Chikungunya, Yellow fever, and Rift Valley fever.

## Introduction

Emerging and re-emerging vector-borne diseases are amongst the prime public health concerns across the world (1). These diseases are not communicable directly among humans instead they do so when favorable condition is formed for the interactions of vectors, animal hosts, climate conditions, pathogens, and susceptible human population (2). Vector-borne diseases are emerging at a growing rate and bearing a disproportionate segment of all new infectious diseases, the vast majority of them being viruses. Several vector-borne pathogens have colonized new regions in the past two decades, while an equivalent number of endemic diseases showed an increase in incidence. Although the introductions and appearances of endemic pathogens are usually regarded as different events, several endemic pathogens are essentially spreading at a limited level synchronized with habitat change. Previous studies reported that vector-borne human infectious diseases like yellow fever and dengue exhibited a wider range of distribution (3).

The emergence of vector-borne diseases appears to be associated with climate factors and climate change though still controversial. Global warming is unequivocal, and every aspect of nature is influenced by climate (4). The impact of climate change is of significant importance in the 21st century. Some previous studies estimated that average global temperatures will have ascended by 1.0–3.5°C by 2,100 (5), escalating the probability of various vector-borne diseases. The effect of the changes in the weather and climatic conditions have direct impact on vectors and the transmission patterns of the pathogens.

Human activities like construction of dams and irrigation schemes can enhance food and energy demands of the developing countries nevertheless they have been concerned for steady transmission amplification or introduction of new vector-borne infectious diseases (6, 7). For instance, ecological modifications connected with water resource expansion and influx of population can facilitate the spread of schistosomiasis to non-endemic areas (8, 9). Recognizing how once dominant vector-borne diseases are reappearing is critical for managing the damage they cause, since the globe is regularly facing challenges from infectious diseases, either receive less attention or are neglected. Therefore, constant awareness of infectious diseases and advances in control efforts is needed to stimulate proper public health reactions (10). Hence this paper aims at reviewing emerging and re-emerging major vector-borne infectious diseases and the challenges for their control.

## Determinants of Emergence and Re-emergence of Vector-Borne Diseases

Emergence and re-emergence of infectious diseases occur over time. Before leading to an outbreak, pathogens pass via different adaptation stages to coup-up with a new host (11). The interplays among pathogens, hosts, and the environment strongly influence the emergence or reemergence of infectious diseases. Moreover, a number of factors such as ecological, environmental, or demographic may contribute to this adaptation and consequent of disease emergence. These factors influence by creating favorable conditions thereby increasing contact with a previously strange pathogen or its natural host or facilitate spreading. Coupled with the progressive evolution of viral and microbial variants and the issue of drug resistance, these factors facilitate for regular emergence and perhaps increase of infection. Spread and establishment of emerging or resurging pathogens can be perpetuated by multifaceted driving forces: climate and non-climate factors (2).

### Climate Change and Climate Factors

Climate change refers to long-term shifts in weather conditions and patterns of extreme weather events. It is part of the many interacting determinants for vector-borne infectious diseases. The impact of climate change on the incidence, transmission season, duration and spread of vector-borne diseases characterizes a serious problem (12). Climate change can affect pathogens directly, through influencing the survival, reproduction, and life cycle of pathogens, or indirectly, by controlling the habitat, environment, or competitors of pathogens and via altering the contact patterns of human-pathogen and human-vector.

Direct effects of temperature on the duration of the extrinsic incubation period of pathogens in insect vectors are crucial in determining whether or not insect-borne diseases can persist or not. Apparently, a pathogen requires a typical range of temperature for their survival and development. Similarly, vector development and survival too is considerably influenced by temperature factors (13). For instance, the two thresholds, maximum temperature of 22–23°C for mosquito development and minimum temperature of 25–26°C for Japanese Encephalitis Virus (JEV) transmission, play decisive positions in the ecology of JEV (14, 15). The development and replication of pathogens transmitted within vectors, the extrinsic incubation period or in the environment also occurs faster at high temperatures (16). Hence, the direct impact of climate change on habitat, and therefore ecosystem change, combined with escalating human encroachment on the natural environment, is adversely limiting biodiversity thereby impacting the emergence and transmission of infectious diseases (17).

Studies showed that though risks from vector-borne diseases are intrinsically susceptible to changes in weather and climate (18, 19), the controversies around impacts of climate change on them focuses on the level to which weather and climate affect their occurrence and intensity vs. human's efforts to control pathogens and their vectors (20). On the other hand, several vector-borne diseases of public health importance are zoonotic being maintained by wildlife, and their occurrence is intrinsically less influenced by man's control efforts (21). Though they are not the most commonly noted reasons for emergence, climatic and weather related factors are explicitly implicated in the emergence of some vector-borne zoonotic diseases. Others reported that land use changes were the most common drivers for their emergence, accounting for 26% of all vector-borne zoonotic diseases followed by unspecified or unknown drivers and international trade and commerce accounting for 14 and 11%, respectively. However, climate and weather related factors were reported to account for 10% as a driver of those diseases (22).

### Non-climatic Factors

Factors other than climatic factors have been implicated to have significant impact on the emergence and/or re-emergence of vector-borne diseases. Some of the major non-climatic factors are global human populations and urbanization, international trade and travel, intensive livestock keeping systems and expansion and modernization of agricultural practices, proliferation of reservoir populations, and antimicrobial drug use (23–25). The association between infectious diseases and socio-political, and economic change from the past to the present has been well-established and complex social and environmental risk factors have been reported in the occurrence of new infectious diseases including vector-borne (26). The appearance of these diseases and re-appearance of the existing ones may indicate that various changes are ongoing in human ecology including rural-to-urban influx of people resulting in crowded urban peripheries, widespread deforestation and war and conflict disrupting social capitals.

Besides host and environmental factors, changes or mutation in the genome of a pathogen, which occurs following exposure to chemicals and antimicrobial agents may lead to gene damage (27) and emergence of drug resistant pathogen variants that could cause new disease (28). Specific processes such as gene mutation, genetic recombination, or reassortment as well as factors that influence microbial agents to change reservoir hosts constitute opportunities for infectious agents to evolve and adapt to new hosts for ease of spread in new ecological niches (28).

#### Globalization

A complex number of factors relating to human behavior and activities, pathogen evolution, poverty, and changes in the environment as well as dynamic human interactions with animals were reported to be linked with appearance and spread of infectious disease (29). The dynamic processes responsible for driving changes in community structure and thus pathogen dynamics at various interfaces eventually seem to favor the appearance and re-appearance of infectious diseases. Nowadays, there are concerns that globalization is impacting on the epidemiology of vector-borne infectious diseases. Many vector-borne viral diseases are becoming threat worldwide (30, 31) owing to territorial expansion of vectors and viruses through globalization and urbanization (32).

#### Urbanization

High influx of rural-to-urban migration has been linked to high-density peri-urban slums which create conducive atmosphere breeding of vectors. Unplanned urbanization is typically associated with poor housing and absence of basic services, including water and waste management, which creates ideal habitats for expansion of vector populations (33, 34). Recent studies suggested that urbanization can facilitate various wildlife-livestock-human interfaces demonstrating a decisive point not only for possible occurrence of new pathogens but also for cross-species spread (35). They added that urbanization also contribute for the emergence of zoonoses, through exploring the ecological complexity of wildlife-livestock-human interfaces. The cross-species spread and onward transmission may indicate a natural response to the evolutionary pressures of pathogen ecology (36). Possible route of transmission can be through food and animal products including meat and dairy products as well as water and waste.

#### Agriculture and Development Projects

Human-induced ecological changes have had their share for the origin of new infections or re-appearance of the existing ones partly by agricultural activities and development projects. Several studies reported that infectious diseases and human-induced land-use changes in agricultural practice are significantly associated (37–40). For instance, encroachment on the natural ecosystem and wildlife by agricultural and land uses expose people and their domestic animals to a wider range of vectors.

Similarly, the expansion of irrigated farming proportionally increased outbreaks of vector-borne diseases. According to reports agricultural drivers are associated with >25% of emerging infectious diseases and >50% of emerging zoonotic infectious diseases in humans (41). Construction of dams, irrigation, and similar development projects were seen to affect vector population densities which in turn determine the occurrence of new diseases and the resurgence of existing ones. For instance, the outbreaks of Rift Valley fever have occurred following the construction of dams and irrigation canals (42).

## Major Emerging and Re-emerging Vector-Borne Infectious Diseases

Historically, human beings have been experiencing huge impacts of vector-borne infectious diseases. Of these, malaria and dengue impose tremendous burden causing an estimated 620,000 and 40,500 deaths in 2017, mostly occurring in Africa and Asia, respectively (43). It is well-known that not all vectors have equal significance with respect to disease transmission. Some vectors have had relatively more impacts than others. A good example of mosquito-borne new and old diseases include those such as malaria, Zika virus fever, dengue, West Nile fever, Crimean-Congo hemorrhagic fever, chikungunya viral disease, Yellow fever, Japanese encephalitis, and Rift Valley fever. These infectious diseases have been posing significant health problem worldwide. In this section emphasis will be given to some of the commonly emerging and re-emerging vector-borne infectious diseases.

### Malaria

Malaria is a vector-borne disease caused by five genera of *Plasmodium* namely *Plasmodium falciparum, P. vivax, P. ovale, P. malariae* and *P. knowlesi* and transmitted by the female Anopheles mosquitoes (44). According to World malaria report of 2018 about 219 million and 435,000 malaria cases and deaths, respectively were recorded in 2017. Approximately 80% of worldwide malaria-related mortality in 2017 occurred in 17 countries in the WHO African Region.

It is well-established that malaria transmission is influenced by environmental factors such as topography, rainfall, climate, and socio-economic conditions of the population. For this reason, tropical regions with warm temperature, heavy rainfall, high humidity and low altitudes are conducive for mosquito breeding, longevity and parasite sporogony. Reports showed that a shift in the altitudinal distribution of malaria toward higher altitudes in warmer years has been observed in Colombia and Ethiopia implying that, in the absence of intervention, the malaria burden will increase at higher elevations as the climate warms (45).

Malaria is a disease of poverty, and contributes to national poverty via limiting foreign direct investment, tourism, labor productivity and trade (46). Studies reported that the cost of illness, treatment and premature death due to malaria was at least USD$12 billion per year in Africa alone thereby promoting poverty (47). Malaria constitutes the top 10 causes of morbidity and mortality among the sub-Saharan Africa in general and Ethiopia in particular.

### Dengue

Dengue is caused by a virus of family Flaviviridae, genus Flavivirus and transmitted by mosquitoes, is broadly spread in tropical and subtropical regions. It is one of the oldest diseases perhaps first documented in Chinese medical encyclopedia in 992. The disease became familiar mainly after the expansion of global shipping industry and port cities in the 18th and 19th centuries. Later on, studies showed that dengue fever was brought to the Americas in conjunction to the slave trade during which the infected slaves from Africa perhaps introduced the infection to the Americas (48). Dengue virus is a prime example of how the interaction between rapid pathogen evolution, human movement, and changing vector ecology has driven emergence (49, 50).

Epidemiological profile of dengue showed an increasing trend in endemic countries. According to recent reports, dengue is the most rapidly spreading arbovirus in the world which threatens a half-billion people globally (51, 52), with dramatical rise in incidence in the urban and periurban areas of Americas (53). Previous studies also showed that the largest dengue epidemic was reported in China in 2014, (54) and the first local dengue case report was from Japan before 70 years in 2014 (55). It has been reported that age-standardized DALY rate increased from 2007 to 2017 for dengue, which made it the sole key vector-borne disease showing a sharp increase by 26% worldwide (43). From the trend it can be evident that a continuous transmission of the disease since the 1950s, aggravated by urbanization, globalization and unsuccessful vector control ending in increased infection and transmission of the virus (32).

### Chikungunya

Chikungunya, which means “disease that bends up the joints” in the Tanzanian Makonde dialect, is a mosquito-borne viral disease caused by an alphavirus from the *Togaviridae* family (56, 57). The origin of the Chikungunya seems controversial as some reports say that it was first isolated from the serum of a febrile human in Tanganyika (Tanzania) in 1953 (58) and others from patients with fever, severe joint pain, and skin rash in Uganda in 1959 (56, 57).

Chikungunya has evolved rapidly after its successful introduction to new localities and getting new anthropophilic vectors. Recurrence of chikungunya virus is a serious public health concern since the virus has been associated with several episodes of epidemics in Africa, Asia, and India (59). Previous studies confirmed that the Chikungunya virus was attributable for the outbreak disseminated across the Indian Ocean which was originated in coastal Kenya during 2004 (60). Even from recent track records, chikungunya seems on the rise as sporadic epidemics were identified in Italy (61) in 2007 and 2017, the first local transmission of the virus in the United States in 2014, (62) and latest outbreaks in the Caribbean, Central and South American regions (63).

### Crimean-Congo Hemorrhagic Fever

Crimean-Congo hemorrhagic fever virus (CCHFV) is an RNA virus caused by a Nairovirus of the Nairoviridae family. Since its first description in Crimea by 1944 the disease has been reported from over 30 countries in Africa, Europe, Asia, and the Middle-East (64). More specifically, healthcare-related CCHFV infections have been reported in several countries worldwide including South Africa (65), Iran (66), India (67), and Turkey (68). CCHF is considered endemic in areas of Southwest Europe as suggested from the seropositive rates of detected animals (69). Portillo et al. (69) also reviewed that positive human cases diagnosed by molecular methods have been reported in Spain from 2016 to August 2020. Similarly, in 2016 the disease was reported in western Spain which appeared to be nosocomial origin as of 2016 (70).

CCHFV is transmitted by Ixodid ticks of the genus *Hyalomma*. Moreover, transmission among humans is also possible via contaminated blood or tissues of infected patients (71). Several reports showed that CCHF has been of major threat to the public health of the people in general and the healthcare workers in particular in resource-poor countries. The case fatality rate of CCHF varies from 5 to 50%; however, in some outbreaks, significant mortality rate of 73% was also recorded (71, 72) which can be influenced by experience of the treatment center in early diagnosis, treatment of the disease and its fatal complications. It has been suggested that Crimean-Congo hemorrhagic fever was included among the priority zoonotic diseases for control.

### Japanese Encephalitis

Japanese encephalitis (JE), major cause of viral meningo-encephalitis in the world, is caused by a single-stranded RNA virus belonging to the genus Flavivirus (73). It was first described in Japan in 1870s and spread throughout huge parts of Asia (74). JE is endemic in different parts of the world mainly Asia extending from Japan to India and Pakistan. Griffiths et al. (74) added that close to half of the population of the world in 24 countries showed endemicity for JE. Transmission of JE virus between animals occurs by Culex mosquitoes throughout eastern and southern Asia and the Pacific border. Natural transmission is maintained among wild and domestic birds, and pigs.

The grounds for the spread of JE are not well-understood, but the dynamic agricultural practices, such as expansion of irrigation which favors vector proliferation and animal husbandry where animals serve as reservoir host can be suggested. Spreading of JEV into highland areas has been reported from altitude of up to 3,100 m of Tibet in China (75). In general, laboratory examination of the virus in mosquito vectors indicates degree of exposure to the disease, intensity of viral activity and genetic variation of JEV in surveyed areas (35). Despite, the availability of safe and effective JE vaccines, JE is still of public health importance in resource constraint endemic countries. Therefore, case-based surveillance should be established in those countries showing high transmission.

### Lyme Disease

Lyme disease is caused by bacterial spirochete referred to as Borrelia burgdorfer and spread to vertebrates including humans by the bite of commonly named deer ticks (*Ixodes* species). It was first described in 1977 as “Lyme arthritis.” Lyme disease is widely distributed in the Western and since its identification; it showed continuous transmission with increasing cases in the northeastern and north central United States.

It was recognized as an important emerging infection in the late 20th century (76). Several reports showed that the incidence of tick bites and cases of Lyme borreliosis has increased substantially over the past decades. In recent years, ~20,000–30,000 confirmed cases of Lyme disease per year have been reported to the Centers for Disease Control and Prevention (77). Approximately 85,000 cases of Lyme disease are reported annually in Europe and in the Netherlands, annually over one million people report a tick bites, and more than 25.000 cases are diagnosed (78).

Ecological conditions conducive to the disease, and the difficulty of prevention, imply that Lyme disease appears to be a continuing concern to the public health (79). Studies indicate that climate change has exacerbated the expansion range of ticks (80), enhancing the probability of menace of Lyme disease, like in areas of Canada in which the vectors were absent before.

### Rift Valley Fever

Rift Valley fever (RVF) is an acute viral febrile haemorrhagic disease caused by a genus *Phlebovirus* of Bunyaviridae family. It affects domestic animals and humans widely in Africa and in the Arabian Peninsula. The disease was initially reported in Kenya in 1930 (81), and has since appeared as sporadic epidemic in cattle and small ruminants. It is characterized by zoonotic transmission to humans in sub-Saharan Africa. Reports showed that RVF has been regarded as a major risk to the health of humans and animals thereby influencing economy and food security (82). Human infection is initiated by mosquito bites mainly Aedes spp. and Culex spp. (83). In addition, the infection can be transmitted through direct or indirect contact with blood or organ of infected animals. Transmission is also possible during slaughtering or handling infected animals, eating infected uncooked meat or drinking raw milk.

### Schistosomiasis

Schistosomiasis is a parasitic disease caused by trematode worms of the genus Schistosoma categorized as intestinal and urogenital schistosomiasis based on the site where the adult worm inhabits in the final host. Intestinal schistosomiasis is caused by *S. mansoni, S. japonicum, S. intercalatum*, and *S. mekongi* while *S. haematobium* causes urogenital schistosomiasis. They are transmitted by different species of fresh water snails; Biomphalaria species, Bulinus species and *Oncomelania* species, being the vectors for *S. mansoni, S. haematobium* and *S. japonicum*, respectively. Although, distribution of schistosomiasis differs widely for each of the parasites, *S. mansoni* and *S. haematobium* are almost prevalent worldwide. On the contrary, *S. japonicum, S. intercalatum* and *S. mekongi* have been restricted to Asian Pacific, West Africa and South-east Asia, respectively. Schistosomiasis is among the 20 diseases considered as neglected tropical diseases (NTDs) by the World Health Organization (WHO).

Transmission of the various types of schistosomiasis is typically linked with poor environmental and sanitary conditions, often affecting people living in unfavorable socioeconomic conditions (8, 84). Calasans et al. (84) reported that there seems a change and even increase in the geographic and seasonal distribution of Schistosoma spp. infections owing to human impacts on environment and climate change thereby making favorable conditions for the disease transmission. In this regards, over the past years transmission of the disease foci in peri-urban and urban areas showed a rising trend (85). Influxes of rural to urban in poor and average-income nations coupled with fast and unplanned urbanization appear to such phenomena (86). More recent study reported that schistosomiasis, being common in urban and urban peripheries, was a significant menace to the public health (87).

### West Nile Fever

West Nile viral (WNV) infection is caused by West Nile virus of the genera *Flavivirus*. It is transmitted principally by *Culex* species of mosquitoes. As opposed to the majority of arboviruses, which are partially or entirely host-specific, WNV is known to have wide range of mosquito vectors and hosts including birds, horses, and other mammals. Such host elasticity might have contributed a lot for humans infection. The clinical features of most human infections are mild and asymptomatic. In more severe cases encephalitis, meningo-encephalitis or meningitis may accompany the disease process.

West Nile fever was first isolated from a febrile patient from the West Nile district of Northern Uganda in 1937 (88). The epidemiology and ecology of the disease was first described broadly by the early 1950s and 1960s when subsequent epidemics took place in the Mediterranean basin (89). Since its first episode in the Americas in 1999, an estimated 7 million cases have been recorded in United States (90). Currently, WNV is regarded among the most important zoonotic diseases of concern to the US population (91). Studies showed that it has been endemic to several parts of Africa, Europe, Asia, Australia, and the Middle East (92). In this regard, despite its global distribution, WNV was not detected in the Americas before the outbreak in 1999.

### Yellow Fever

Yellow fever is caused by a yellow fever virus belonging to the genus of Flavivirus in the family of Flaviviridae. It is transmitted by the bite of infected *Aedes* species of mosquito. Yellow fever is among viral hemorrhagic fevers and the most devastating disease in history. The virus was originated in Africa and transmitted to the western hemisphere during the time of slave trade. The first outbreak was reported in 1648 in the Yucatan Peninsula (93) and when they happen, the outbreaks have been characterized with high mortality (94). Since its emergence, yellow fever epidemics have been persistent in South America and Africa, affecting the urban and rural populations indiscriminately. According to recent reports, epidemics of Yellow fever in urban areas of some African countries like Angola and Democratic Republic of Congo showed an increasing trend and imported cases were reported in China and Kenya (95). The rising of international travel and population movements aggravated for the pushing of the disease into large metropolitan parts in tropical and subtropical areas (96). Previous WHO reports showed that 47 countries in Africa and Central and South America were regarded as endemic for YF with estimated annual burden of 200,000 and 60,000 severe cases and deaths, respectively (97). Currently, vaccines against flaviviral infections of humans are available for yellow fever.

### Zika Fever

Zika virus (ZIKV) was first isolated in the Zika forest in Uganda from a rhesus macaque by 1947 (98). It is one of the members of flaviviruses responsible for important human diseases. Zika is a mosquito-borne disease often referred to as Zika fever. Zika virus was emerged in South and Central America in Southern Florida and at the Texas/Mexico border in the USA in 2015 (99).

The outbreak of Zika virus illness was reported in Yap Island, Federal state of Micronesia which indicates the transmission of the disease outside Africa and Asia (100). Toward the end of 2013, imported cases from French Polynesia were reported in New Caledonia and cases of autochthonous transmission were reported in January 2014 (101, 102). This suggested that ZIKV was introduced to the Americas from any of the Pacific Islands such as French Polynesia, New Caledonia, Easter Island, or Cook Islands. So far, there is no vaccine candidate approved for Zika fever. It has been reported that Zika virus has resulted in serious epidemics in the American regions in 2015 (103). By the same year an epidemic of ZIKV stroke Brazil and then disseminating to South and Central American and the Caribbean countries. In the following years, the disease was spread in limited nations of Africa and Southeast Asia.

## Zoonosis and Vector-Borne Infectious Diseases

Zoonosis and the emergence of zoonotic diseases are complex problem influenced by several interlinking factors. Vector-borne zoonotic diseases are emerging at an increasing rate compared to directly transmitted ones. Previous studies reported that a high number of emerging infectious diseases are vector-borne for over 15 years old (104). Although 14% of human infectious diseases are vector-borne, vector-borne zoonotic diseases account for 22% among the whole newly arising diseases of humans implying their disproportionate representation (104). Similarly, it is estimated that 60 to 80% of newly arising infections are zoonotic in origin and initially animals served as reservoir for their survival (104). Jones et al. (104) added that at least 70% of these emerging zoonotic diseases have had their origin from wildlife with inter-species spread.

Since the 1940s the rate of zoonotic disease emergence has shown increasing trend as designated by the rising incidence of emerging infectious disease events, even after effective intervention for increasing infectious disease (104). Previous reports showed that some of the zoonotic arbovirus diseases such as Rift Valley fever virus, Japanese encephalitis virus, Dengue virus, and Chikungunya virus expanded in global distribution in the past decade (105). Similarly, WNV is regarded among the major zoonotic diseases of public health menace in the USA (91). It has been projected that, almost one billion public are at risk of contracting the virus transmitted by the mosquito of *Aedes* spp by the next century (106).

## The Burden of Vector-Borne Infectious Diseases

Vector-borne infectious diseases pose a huge burden of morbidity and mortality worldwide, particularly affecting the resource scarce and economically lower segments of the society. Although their impediment is amplified across the world, the morbidity and mortality they inflict is highest in tropical and subtropical areas. Even among the tropics and subtropics they disproportionately pose higher burden impoverished population. It has been noted that vector-borne infectious diseases account for 17% of estimated global burden of all infectious diseases (107).

The global impact of the vector-borne diseases is further intensified by the recurrent emergence of new, unrecognized, and re-emergence of the existing outbreaks. Over the past few decades at least 30 new infectious agents affecting humans have emerged, most of which are zoonotic and their origins were correlated significantly with socioeconomic, environmental, ecological and climatic factors.

Vector-borne infectious diseases impose an important global burden on public health increasing health inequalities. The plague of newly appearing infectious diseases is already well-documented since ancient times. Vector-borne infectious diseases were known to be the most devastating pandemic in the human history. One counter example such notorious vector-borne disease which claimed the life of 25-40 million is bubonic/pneumonic plague (10).

## Control Measures and Challenges of Vector-Borne Infectious Diseases

Several tools and control approaches have been practiced to mitigate emerging and reemerging vector-borne infectious diseases. Intensifying active surveillance, quality assured early diagnosis and effective case management has been of paramount significance. Molecular approaches like genome sequencing and phylogenetic tracing studies can play a decisive part in precisely identifying the novel pathogens (108).

The control of vector-borne diseases is among the major challenges across the world health program. The current fast and uncontrolled urbanization has intensified the concern in resolving these problems using structured strategic plans which can be designed and implemented at global and local stages. The high prevalence and rise in incidence of endemic vector-borne diseases alarmed stakeholders for successful control and treatment of victims with related episodes. Accordingly, the global burden of infectious diseases including vector-borne ones showed considerable decline during the past decades owing to the advancement of modern medicine, poverty reduction approaches and socioeconomic development, and the use of more efficient intervention and control measures (109). Among the popular and efficient vector control approaches was use of chemical insecticides. Despite the success stories of chemical insecticides, insecticide resistance has emerged as a major threat to vector-borne control that mainly depends on targeting vector populations (110). Nevertheless, advances at an international scale covers significant successes gained regionally.

It has been well-noticed that several vector-borne diseases are zoonotic and their transmission frequency in vectors is facilitated by wildlife reservoirs. Such phenomenon of circulating between vectors and animal reservoir hosts has become a bottleneck for their control and interventions. Perhaps as a result such complex interface among vector-pathogen-host, major zoonotic arbovirus diseases showed huge expansion in global distribution in the past decade (105).

As of today, though diverse control and intervention approaches are at our disposal, most of the control programs are still further challenged by one or multiple variables such as environmental change, insecticide resistance, population growth and urbanization and climate change. Hence, as shortage of funding and weak programmatic capacity prevail, a prompt need for enhancing partnership and collaboration is required for empowering the capacity for surveillance and control new vector control tools (111). Financial constraint also prevails in advancing development and testing of novel pathogen diagnostic tools.

To sum up, vector control has been the key tool in fighting against vector-borne diseases so far and still greatly effective, provided that it is broadly implemented. Actually, it remains the sole and appropriate control tool available at our disposal for many diseases including vector-borne ones.

## Concluding Remarks

Vector-borne infectious diseases impose an important global burden on public health. For over past few decades epidemics of vector-borne emerging diseases were rising perhaps through multiple driving forces including socioeconomic, environmental, global warming and climate change. Collaborative research networks on zoonotic and vector-borne emerging and re-emerging infectious diseases remain the most crucial in addressing the root problems for long-term plan. In this regard, empowering One Health approach encompassing public health experts, veterinarians, entomologists, and parasitologists should be prioritized. Besides, the role of international donors and fund raising agents should be give due attention. Nowadays, numerous emerging, re-emerging, and stable vector-borne infectious diseases are becoming well-managed yet the future efforts on blocking the emergence of new diseases seem uncertain. This may alert for uninterrupted fight against emerging vector-borne infectious diseases.

## Author Contributions

Both authors listed have made a substantial, direct and intellectual contribution to the work, and approved it for publication.

## Conflict of Interest

The authors declare that the research was conducted in the absence of any commercial or financial relationships that could be construed as a potential conflict of interest.

## Publisher's Note

All claims expressed in this article are solely those of the authors and do not necessarily represent those of their affiliated organizations, or those of the publisher, the editors and the reviewers. Any product that may be evaluated in this article, or claim that may be made by its manufacturer, is not guaranteed or endorsed by the publisher.
